# Supplementary material for: Utilizing CNNs for classification and uncertainty quantification for 15 families of European fly pollinators
Source: PLoS One. 2025 Sep 10;20(9):e0323984. doi: 10.1371/journal.pone.0323984 (PMC12422425; doi:10.1371/journal.pone.0323984)
Supplement: Attachment — Submitted filename: Letter_to_reviewers_PONE-D-25-20622.pdf [file pone.0323984.s001.pdf]

# Response to the reviewers

## Title: Utilizing CNNs for classification and uncertainty quantification for 15 families of European fly pollinators

## Manuscript Number: PONE-D-25-20622

Dear Irene Nathalie Fernandez Tolentino,
Dear Editors,

Thank you very much for considering our manuscript for publication PLOS ONE subject to the requested revisions based on reviewers' comments. Please find below a point-by-point answer to the comments (***bold, cursive and bullet points)*** in blue and new text which was added to the main manuscript in red.

We acknowledge the valuable reviewers' comments and hope that the revised manuscript meets all specifications with respect to the changes made.

Sincerely,

Thomas Stark

## Reviewer #1:

*This is a very well written and presented manuscript; discussion and conclusions are well thought through, and relevant literature used. It will be of interest to many others regarding methods and overall conclusions.*

- *(Line 168) How did the bounding box but also the preprocessing get done, what software environment?*

Thank you for bringing this to our attention we added more detail of making the bounding boxes and expanded the preprocessing process in more detail.

The bounding box generation was conducted in a two-step process. First, we used a pre-trained object detection model based on the approach described in [23] to automatically generate initial bounding boxes around the Diptera specimens. In a second step, these boxes were manually refined by two expert ecologists to ensure high annotation accuracy. This manual correction was performed for all ~30,000 images using the open-source tool LabelImg within a Python-based environment. This combination of automated detection and expert validation ensured both efficiency and high-quality ground truth for subsequent model training.

(L.213-218) In Figure 1 our methodological approach is illustrated in detail. Initially, bounding boxes are created for 29,374 images to crop them, ensuring that only the entire body of the Diptera is visible while removing any background as seen in Figure 1(A). Bounding Boxes were

first generated using a pre-trained object detection model from [23] and then manually corrected by two expert ecologists using the open-source tool LabelImg in a Python environment.

We are not entirely sure whether the preprocessing refers to image preprocessing or data selection preprocessing. Nevertheless, for image preprocessing, we applied z-score normalization using the RGB mean and standard deviation values from ImageNet, which we also used for pretraining our three CNN models. This normalization helps standardize pixel distributions across images and is commonly used in deep learning pipelines to improve model convergence and performance [32,37,38]

Regarding dataset curation, this was a more involved step due to the high variability of GBIF images. We invested considerable effort into selecting images that depict adult Diptera on flowers or in natural environments, while excluding larval stages, museum specimens, or highly artificial backgrounds. While GBIF image tags were partially helpful for filtering, most of this refinement took place during the bounding box creation and correction process, where we manually removed unsuitable samples to ensure a consistent and high-quality dataset.

- *Fig3 misleading legend, should say "With" and "Without" (rather than False/True), the figure can be interpreted differently with the words False/True*

Thank you for highlighting this typo, we fixed it in the revised manuscript.

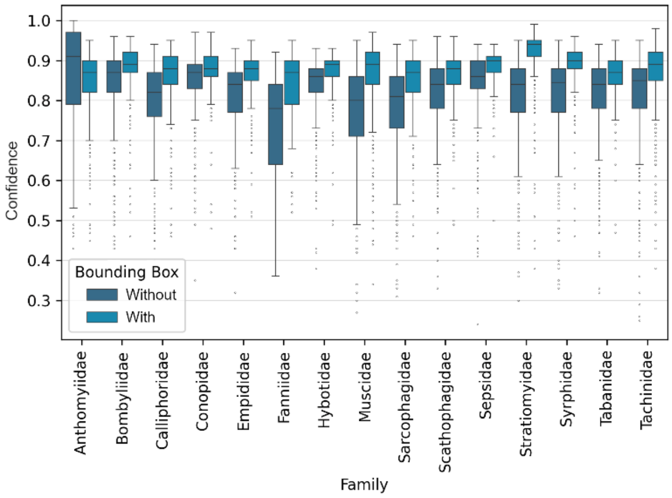


- *Fig 3 caption – spelling "boundinx", should be Bounding*

Thank you again for highlighting this typo, we fixed it in the revised manuscript.

**Fig 3. Confidence values** for EfficientNetB4 for all 15 Diptera families for correct classification results with and without cropping its Bounding Boxes.

- *The abstract states "Our dataset comprises a wide range of morphological and phylogenetic features, such as wing venation patterns and wing shapes" but this is misleading; the images used were general habitus images of the body. Yes "features of the wings" are part of this general body but they are not the only feature. The statement in the abstract implies wing features are the central part of the images.*

Thank you for highlighting this point. We agree that the original phrasing may have been too strong and will soften the wording in the abstract accordingly.

Pollination is essential for maintaining biodiversity and ensuring food security, and in Europe it is primarily mediated by four insect orders (Coleoptera, Diptera, Hymenoptera, Lepidoptera).

However, traditional monitoring methods are costly and time consuming. Although recent automation efforts have focused on butterflies and bees, flies, a diverse and ecologically important group of pollinators, have received comparatively little attention, likely due to the challenges posed by their subtle morphological differences. In this study, we investigate the application of Convolutional Neural Networks (CNNs) for classifying 15 European pollinating fly families and quantifying the associated classification uncertainty. In curating our dataset, we ensured that the images of Diptera captured diverse visual characteristics relevant for classification, including wing morphology and general body habitus. We evaluated the performance of three CNNs, ResNet18, MobileNetV3, and EfficientNetB4 and estimated the prediction confidence using Monte Carlo methods, combining test-time augmentation and dropout to approximate both aleatoric and epistemic uncertainty. We demonstrate the effectiveness of these models in accurately distinguishing fly families. We achieved an overall accuracy of up to 95.61%, with a mean relative increase in accuracy of 5.58% when comparing uncropped to cropped images. Furthermore, cropping images to the Diptera bounding boxes not only improved classification performance across all models but also increased mean prediction confidence by 8.56%, effectively reducing misclassifications among families. This approach represents a significant advance in automated pollinator monitoring and has promising implications for both scientific research and practical applications.

- *Early discussion (lines 305 +) regarding the time to make bounding boxes; unsure if the authors know of Roboflow (www.roboflow.com) - it has some semi automated features and is very easy to use.*

Thank you for this helpful suggestion. We were only loosely aware of Roboflow but had not used it in our workflow. Its semi-automated features indeed seem very helpful, and we now mention in the discussion that such tools could support more efficient annotation in future work.

(L.375-388) Creating bounding boxes manually is an extremely time-consuming process. For the 29,374 images used in this study, bounding boxes had to be created by experts, following a consistent workflow to ensure that each specimen is cropped uniformly. This task requires not only significant time but also specialized expertise to accurately delineate the objects of interest. The labor-intensive nature of this manual approach underscores the potential benefits of automation. Also semi-automated labeling tools, could further support and streamline this process. It would be very interesting to explore the use of object detection algorithms to automatically crop images similar to the methods used in [49] and [25].

- *One thing the authors could examine in any future work is the ranges and overlap of confidence scores of those images that are scored correctly vs incorrectly. There is some discussion of this on lines around 380-390. This connects with "trustworthiness" that the authors mention in the introduction. It is an issue that we are facing in our lab group - where is there overlap in scores between right and wrong, and so it is difficult for the user to be confident of the score.*

Thank you for this very interesting suggestion. We have also noticed similar issues in our results and agree that analyzing the overlap in confidence scores between correctly and incorrectly classified images is a promising direction. This is definitely a topic we will look into more closely in future work, especially now knowing that other groups are encountering a similar challenge.

## Reviewer #2:

*I enjoyed reading this study and there were some interesting methodology and results however some information was lacking. Further details are required in the materials and methods for transparency and the figure captions need fuller explanations so that if viewed independently the reader understands them. A key aspect of this study are the uncertainty quantification and mean confidence calculations; these provide a potentially useful metric for researchers using machine learning classifiers however some methodological details are missing.*

- *There is also some imbalance in the number of images for each of the families and I would be interested to see the f1 scores to determine whether this has impacted CNN performance.*

Thank you for your valuable observation regarding potential class imbalance and the relevance of F1 scores. While we did observe some variation in the number of images per fly family, we do not consider the imbalance to be substantial enough to significantly skew model performance. To evaluate this further, we did compute the macro-averaged F1 scores alongside other metrics. However, we chose to focus on overall accuracy and Cohen's kappa in the manuscript to maintain clarity and avoid redundancy, as the F1 scores were very similar to these primary metrics.

Nevertheless, we appreciate your suggestion and have now included a table comparing accuracy, kappa, and F1 score per model and variant. As shown in the table below, the F1 scores follow the same trends as the accuracy and kappa metrics. For example, the EfficientNet model with bounding box cropping (BB) achieved the highest values across all three metrics (Accuracy: 95.61%, Kappa: 0.9590, F1: 0.9622), while the noBB variants consistently performed lower. These results support our interpretation that minor class imbalance did not substantially impact model performance.

| *Model* | *Variant* | *Accuracy* | *Kappa* | *F1* |
|---|---|---|---|---|
| *MobileNet* | noBB | 88.78% | 0.8887 | 0.8963 |
| *ResNet* | noBB | 88.58% | 0.8895 | 0.8973 |
| *EfficientNet* | noBB | 90.35% | 0.9044 | 0.9110 |
| *MobileNet* | BB | 93.87% | 0.9401 | 0.9444 |
| *ResNet* | BB | 93.16% | 0.9384 | 0.9426 |
| *EfficientNet* | BB | 95.61% | 0.9590 | 0.9622 |

- *Abstract: I would like more information about the methodology here and some more results – e.g. what method did you use to determine accuracy and prediction confidence? What was the improvement in accuracy using bounding boxes?*

Thank you for highlighting this important point. We have revised the abstract to include more specific details regarding our methodology and key results. In particular, we now explicitly state that overall accuracy (OA) was used as the main performance metric, and that prediction confidence and uncertainty were quantified using Monte Carlo methods, combining test-time augmentation and dropout to estimate both aleatoric and epistemic uncertainty.

Additionally, we clarified the performance gains achieved through image cropping. Specifically, we now report that EfficientNetB4 reached an overall accuracy of 95.61%, representing a relative improvement of approximately 5.82% over the uncropped version. We also mention that, on average, cropping images to the bounding boxes of Diptera resulted in a 5.58% increase

in classification accuracy and an 8.56% increase in prediction confidence across all models. These additions help emphasize the benefit of expert-defined cropping in boosting model reliability.

We hope these clarifications improve the completeness and transparency of the abstract.

Pollination is essential for maintaining biodiversity and ensuring food security, and in Europe it is primarily mediated by four insect orders (Coleoptera, Diptera, Hymenoptera, Lepidoptera). However, traditional monitoring methods are costly and time consuming. Although recent automation efforts have focused on butterflies and bees, flies, a diverse and ecologically important group of pollinators, have received comparatively little attention, likely due to the challenges posed by their subtle morphological differences. In this study, we investigate the application of Convolutional Neural Networks (CNNs) for classifying 15 European pollinating fly families and quantifying the associated classification uncertainty. In curating our dataset, we ensured that the images of Diptera captured diverse visual characteristics relevant for classification, including wing morphology and general body habitus. We evaluated the performance of three CNNs, ResNet18, MobileNetV3, and EfficientNetB4 and estimated the prediction confidence using Monte Carlo methods, combining test-time augmentation and dropout to approximate both aleatoric and epistemic uncertainty. We demonstrate the effectiveness of these models in accurately distinguishing fly families. We achieved an overall accuracy of up to 95.61%, with a mean relative increase in accuracy of 5.58% when comparing uncropped to cropped images. Furthermore, cropping images to the Diptera bounding boxes not only improved classification performance across all models but also increased mean prediction confidence by 8.56%, effectively reducing misclassifications among families. This approach represents a significant advance in automated pollinator monitoring and has promising implications for both scientific research and practical applications.

## Introduction

- *Line 12: A definition of a CNN would be useful here.*

We have added a brief definition of Convolutional Neural Networks (CNNs) directly after their first mention in the text to improve clarity for readers unfamiliar with the concept. Additionally, we now include two standard references to foundational CNN [11, 12] to support this explanation.

(L.10-15) The limited availability of images for flies, compared to more charismatic taxa of pollinators, makes it difficult to create a comprehensive dataset, which is essential for successfully training Convolutional Neural Networks (CNNs) [9, 10]. CNNs are a class of deep learning models particularly well-suited for image recognition tasks due to their ability to automatically learn hierarchical spatial features from raw pixels [11, 12].

- *Line 28: Further expansion of this paragraph would help to highlight the rationale for your study*

Thank you very much for highlighting the importance of clearly positioning our study within the broader context of deep learning applications for arthropod classification. In response, we have revised and expanded the background section to better contextualize our work and explain its rationale. Specifically, we now provide a more detailed overview of how deep learning has been applied to various arthropod groups, citing several recent studies across different taxa. We also added a dedicated paragraph emphasizing that pollinating flies—despite being the world's second most important group of pollinators after bees—have received comparatively little attention in this research area. To support this point, we included several key references [6,27,28,29] demonstrating the ecological and functional relevance of Diptera, especially in

regions or conditions where bees are less prevalent. These additions strengthen the motivation for our study and highlight the gap our work aims to address: the need for robust deep learning models capable of identifying pollinating flies, at least to the family level, for comprehensive biodiversity monitoring and conservation efforts.

(L.32-41) However, pollinating flies have not yet been systematically addressed in this line of research, despite being the world's second most important and abundant pollinators after bees [6,27]. Flies, particularly syrphids and other Diptera, make vital contributions to both natural ecosystems and agricultural systems, especially in high-latitude or high-altitude environments where bee populations decline or shift [28, 29]. Long-term studies have shown shifts in pollinator communities over time, with flies playing an increasingly central role in many regions [29]. Given their ecological importance and diversity, it is crucial that future deep learning models are developed to reliably identify pollinating flies, at least to the family level, to ensure more comprehensive monitoring, support biodiversity assessments, and inform conservation strategies.

- *Line 36: Clarification of uncertainty estimation in relation to previous works and how it applies to this study would be beneficial in this paragraph.*

Thank you for your comment regarding the clarification of uncertainty estimation in relation to previous works. In response, we have revised the paragraph to explicitly highlight how our approach to uncertainty estimation builds upon and differs from prior applications in ecological deep learning. We now clarify its specific relevance to pollinator classification tasks, where visual ambiguity and limited training data are common challenges. Additionally, we have added two references (\cite{Christin2019, Norouzzadeh2018}) that illustrate previous use cases of uncertainty in ecological contexts, providing a clearer connection between our methodology and the broader scientific literature.

(L.42-50) Using image classification techniques for pollinator identification often lacks insight into the confidence levels in its predictions. This is particularly relevant in challenging cases where image quality or species similarity can impact accuracy [19,30]. Ensuring trustworthiness is critical and uncertainties play a crucial role in this case. This study delves into integrating uncertainty estimation methods within deep learning models to enhance predictive trustworthiness by explicitly addressing aleatoric and epistemic uncertainties. We further clarify how these uncertainty estimates compare to previous approaches in ecological deep learning studies and demonstrate their specific value for insect classification, where limited training data and ambiguous visual cues are common [31,32].

- *Line 58: a brief description of your uncertainty quantification methods in this paragraph would emphasize the novelty of your approach*

Thank you for pointing out the need for more detail on our uncertainty quantification methods. We have now included a brief description in the introduction text, explaining that we use a combination of Test-Time Augmentation (TTA) and Test-Time Dropout (TTD), with Monte Carlo sampling to approximate predictive uncertainty. This approach follows the framework introduced by Gal and Ghahramani [35], and allows us to capture both aleatoric and epistemic uncertainty during inference. We believe this addition strengthens the transparency and reproducibility of our approach and aligns it with established practices in uncertainty-aware deep learning.

(L.52-65) The uncertainty estimation goes beyond traditional predictions, providing insight into the confidence and reliability of the model [33]. Incorporating these uncertainty estimation methods is crucial in real-world applications where decision-making about pollination abundance counts is based on accurate and reliable predictions. By transparently quantifying uncertainties, the models not only improve interpretability, but also provide a measure of

confidence in their predictions [34]. This study outlines the methodologies used to capture and quantify uncertainties, contributing to the broader goal of establishing trust in deep learning models for image classification. Specifically, we use a combination of Test-Time Augmentation (TTA) and Test-Time Dropout (TTD), leveraging Monte Carlo sampling to approximate predictive uncertainty, as proposed by Gal and Ghahramani [35]. One key research question is whether CNNs can effectively differentiate between the 15 families of pollinating flies, including a comparison of the impact of provided images versus cropping on classification accuracy.

## Materials and Methods

- *General - Further information about Diptera and their identification features would be interesting, particularly examples of wing venation.*

Thank you for your comment regarding Diptera identification features. We have now expanded the manuscript to include additional morphological traits used for family-level classification, with a particular focus on wing venation. For example, we highlight the presence of the *vena spuria* in Syrphidae and the distinct venation of Empididae, as well as traits distinguishing calyptrate from acalyptrate flies. We also added a note on the relevance of general habitus, supporting the plausibility of CNNs learning visual cues even under limited image quality.

(L.98-112) Ultimately, understanding these nuances is critical to improving the accuracy, reliability, and interpretability of automated identification systems. Further morphological cues, particularly wing venation, provide crucial identification features in Diptera. For example, Syrphidae possess the vena spuria, a vein nearly exclusive to this family, while Empididae also display distinctive venation patterns. Calyptrate and acalyptrate flies may share similar wing venation, but calyptrates can be further distinguished by the presence of vibrissae bristles above the mouth. Additional traits such as body hair (e.g., hypopleural bristles), scutellum shape, and bristle positioning on the thorax and legs also aid in family-level classification. Despite the subtlety of these features, many experienced taxonomists can identify families such as Bombyliidae, Empididae, and Syrphidae by general habitus alone, even from limited image quality—a useful analogy for the capability of CNNs to learn from complex but non-explicit visual cues.

- *Including the formula for the mean confidence score would provide further clarity.*

Thank you for your helpful suggestion. In response, we have added a set of equations (Equations 1–3) to the revised manuscript that formally describe how prediction confidence is estimated using Monte Carlo sampling. These equations illustrate how multiple stochastic forward passes are generated via test-time augmentation (TTA) and test-time dropout (TTD), how the most frequently predicted class (mode) is selected, and how the final confidence score is computed based on prediction consistency. We also included references to Gal et al. (2017) and Wang et al. (2019) to support the use of these techniques. We hope this addition improves the clarity and transparency of our method.

$$y^{(t)} = \arg\max_k f_\theta^{(t)}(\tilde{x}), \quad \text{for } t = 1, \dots, T \tag{1}$$

$$\hat{y} = \text{mode}\left(\{y^{(1)}, y^{(2)}, \dots, y^{(T)}\}\right) \tag{2}$$

$$\text{Mean Confidence}(\hat{y}) = \frac{1}{T}\sum_{t=1}^{T} \mathbb{I}[y^{(t)} = \hat{y}] \tag{3}$$

Equations (1) to (3) describe how we estimate prediction confidence using multiple stochastic forward passes through the model. In Equation (1), we apply random image augmentations and

enable dropout during test time to generate diverse predictions $y^{(t)}$ for the same input image. This process is repeated $T$ times to simulate uncertainty due to both data variability and model limitations [36]. In Equation (2), we identify the most frequently predicted class across all T iterations, known as the mode, which becomes the final predicted label $\hat{y}$. Equation (3) then calculates the confidence score as the proportion of predictions that agree with this final class. This gives a simple but effective estimate of how certain the model is in its prediction, the higher the confidence, the more consistent the model's predictions were under different random conditions [43].

- *Line 79: I think this paragraph would be better placed in the introduction*

Thank you for your suggestion. While we did not move the paragraph into the Introduction as proposed, we have revised the wording to make it more appropriate and precise for the *Materials and Methods* section. We hope this adjustment aligns with your expectations.

(L.98-100) The dataset includes fly families selected to represent a range of morphological differences and evolutionary relationships, providing visual characteristics relevant for training and validating the classification models.

- *Line 147: Which image augmentations were used and in which proportions?*

We added more context on the augmentations used and included Table 3. which groups the augmentations into Basic, Color, Geometry and Mix categories and the percentage chance of them being applied to each image.

(L.172-183)We provide a random set of image augmentations during both the training phase and when the model is applied to the test dataset. This TTA approach ensures that the model encounters a diverse range of augmented inputs during training and testing, facilitating its ability to generalize and accurately assess uncertainty in real-world scenarios [43,44]. The augmentations were grouped into four categories: (i) basic augmentations (horizontal flip and 90° rotation, both with (p=1.00); (ii) color augmentations (jitter, channel shuffle, Gaussian noise, blur, and sharpness, each with (p=0.1); (iii) geometric distortions (thin plate spline, random cropping, and erasing, each with (p=0.1); and (iv) mixing-based augmentation using CutMixV2 (p=0.1). All augmentations were applied stochastically across the ensemble predictions and averaged to obtain final probabilities, following established practices for test-time uncertainty estimation [43].

**Table 3. Overview of data augmentation techniques used during test-time augmentation (TTA) and the percentage value if it is applied (p).**

| Group | Techniques | p |
|---|---|---|
| Basic | Horizontal Flip | 1.0 |
| | Rotation 90° | 1.0 |
| Color | Color Jitter | 0.1 |
| | Channel Shuffle | 0.1 |
| | Gaussian Noise | 0.1 |
| | Median Blur | 0.1 |
| | Sharpness | 0.1 |
| Geom. | Thin Plate Spline | 0.1 |
| | Crop (2×2) | 0.1 |
| | Random Erasing | 0.1 |
| Mix | CutMix V2 | 0.1 |

- *Line 154: What percentage of dropout did you use?*

We added the dropout value we used for both training and testing of 30%.

(L.184-189) Epistemic uncertainty, on the other hand, is rooted in the limitations of the model's knowledge [36]. It reflects uncertainty arising from a lack of understanding or exposure to various data during training. We addressed epistemic uncertainty by incorporating test-time

dropout (TTD). This helps the model recognize when faced with unfamiliar patterns not encountered during training, reducing the uncertainty associated with knowledge gaps [45]. For the dropout method we use the same value of 0.3 as shown in [14].

- *Line 160/161: Did you use augmented data when you trained these models?*

Yes, we used the same data augmentations and percentage values during both training and test-time inference (TTA) as listed in Table 3. This ensured consistency between the training, validation and test time phases.

- *Figure 1: More detail in the caption is needed – are the confidence figures for all species?*

Thank you for your helpful comment. We have expanded the figure caption to provide more detail on the three main steps illustrated: (A) the creation of bounding boxes (using a Fannidae example), (B) the training of CNNs on cropped and uncropped images, and (C) the application of test-time augmentation and dropout for uncertainty estimation. We also added a clarification that, although one species is shown for illustration, the uncertainty quantification was performed across all 15 Diptera families in the dataset. We hope this addresses your concern. As we were not entirely sure what was meant by "confidence figures for all species," we interpreted it as a request to clarify the general scope of the uncertainty analysis, but we're happy to revise further if needed.

**Fig 1. Uncertainty quantification approach.** (A) Creation of bounding boxes around Diptera specimens to generate both cropped and uncropped versions of the dataset. (B) Training of CNNs using both image variants to evaluate the effect of cropping on classification performance. (C) Confidence estimation using test-time augmentation (TTA) and test-time dropout (TTD) with Monte Carlo sampling to assess the model confidence. While this figure shows an example for one Family, Fannidae, the uncertainty quantification was applied across all families in the dataset.

- *Line 197: This paragraph is a little ambiguous, which configurations did you investigate? Learning rate, batch size etc?*

Thank you for pointing out the ambiguity in our original description. We have revised the paragraph to clarify which training configurations were investigated. Specifically, we used 8-bit RGB images resized to 640×640 pixels and normalized using z-score normalization. The CNNs were trained using the AdamW optimizer with a fixed learning rate of 0.001 and a CosineAnnealingWarmRestarts scheduler. We trained for up to 100 epochs with early stopping, using cross-entropy loss with a label smoothing factor of 0.1. These configurations were selected to explore the tradeoff between per-epoch training time and overall convergence behavior. The revised paragraph now explicitly details these settings.

(L.244-254) In this study, we systematically evaluated the tradeoff between the per-epoch training speed of a CNN and the total time required to reach optimal validation performance. Specifically, we investigated how various training settings affect convergence and generalization. Our experiments used 8-bit RGB images resized to 640×640 pixels and normalized using z-score normalization based on ImageNet statistics. The models were trained for a maximum of 100 epochs using early stopping, with cross-entropy loss incorporating label smoothing (0.1) and the AdamW optimizer. A fixed learning rate of 0.001 was combined with a CosineAnnealingWarmRestarts scheduler to allow for dynamic adjustment during training. By keeping these settings consistent, we aimed to strike a balance between fast per-epoch training and reliable generalization to unseen data, while minimizing the risk of overfitting.

- *Experimental set up: Information regarding the performance metrics and how they are calculated is missing. The Kappa score is calculated but not explained.*

We thank the reviewer for the suggestion. In response, we have now added a clear explanation of how accuracy and the Kappa score are computed. Specifically, we included equations using true positives (TP), true negatives (TN), false positives (FP), and false negatives (FN) to define overall accuracy and Cohen's Kappa coefficient. We also provided the equation for the expected agreement by chance (pep_epe) used in the Kappa calculation. These equations are now included in the manuscript as Equations (4)–(6) to improve clarity and reproducibility.

(L.264-272) Table 4 compares three CNNs in terms of accuracy and efficiency, detailing their number of parameters, overall accuracy (OA), Kappa score, mean confidence, training epochs, time per epoch, test time for 100 Monte Carlo iterations, and whether the image was cropped to its bounding box. OA and Kappa are calculated as shown in Equations (4) and (5), where the Kappa score incorporates the expected agreement by chance (6) to provide a more robust evaluation for imbalanced datasets, mean confidence is calculated using equation (3).

$$\mathrm{OA} = \frac{TP + TN}{TP + TN + FP + FN} \quad (4)$$

$$\kappa = \frac{p_o - p_e}{1 - p_e} \quad (5)$$

where $p_o$ = Accuracy is the observed agreement, and $p_e$ is the expected agreement by chance, defined as:

$$p_e = \frac{(TP + FP)(TP + FN) + (FN + TN)(FP + TN)}{(TP + TN + FP + FN)^2} \quad (6)$$

## Results

- *Line 217: I think you are understating your results a little here, all your models achieved OAs in excess of 88% and mean confidence of more than 78%. The Kappa score could also be mentioned here.*

Thank you for your valuable suggestion regarding the inclusion of additional evaluation metrics. In response, we have now explicitly incorporated the Kappa score alongside overall accuracy (OA) to provide a more robust assessment of model performance. As Kappa accounts for agreement beyond chance, its inclusion strengthens the reliability of our evaluation. The Kappa results are now discussed in the results section and included in Table 4, showing consistently high agreement levels across all models and confirming the effectiveness of our classification approach.

(L.291-300) A similar pattern is observed in the Kappa scores, which provide a more robust measure by accounting for chance agreement. MobileNetV3 Large improves from a Kappa of 0.8887 (uncropped) to 0.9401 (cropped). ResNet-18 increases from 0.8895 to 0.9384, and EfficientNetB4 from 0.9044 to 0.9590. These high Kappa values further confirm that the classification performance is not only accurate but also consistent across the different models and image configurations.

- *Line 232 and 235: You could add the mean improvement of OA and mean confidence across all CNNs for further clarity.*

We appreciate the reviewer's suggestion to provide a more compact and comparative view of model improvements. To address this, we have calculated and added the mean overall accuracy, Kappa score, and mean confidence across all three CNNs for both the uncropped and cropped image sets. This summary clearly illustrates the benefits of bounding box cropping, with all metrics showing notable improvements. The averaged results are now described in the manuscript to highlight the consistent gains in performance and confidence across models.

(L.301-307) Averaging across the three CNN architectures, the use of cropped images leads to a clear improvement in all performance metrics. The mean overall accuracy increases from

89.24\% (uncropped) to 94.21\% (cropped). Similarly, the mean Kappa score rises from 0.8942 to 0.9458, indicating improved agreement beyond chance. The mean confidence also shows a substantial boost, increasing from 78.98\% to 85.74\%. These results highlight that cropping images to their bounding boxes not only improves classification accuracy but also enhances model certainty and reliability.

- *Figure 2: I would include the percentages only in the confusion matrices to make the figure more readable*

Thank you for the suggestion regarding the readability of the confusion matrix figures. We have revised the figures to improve clarity. Specifically, we now display only the percentage values along the diagonal to represent correct classifications, while retaining both the percentage and the absolute number of misclassifications in the off-diagonal cells. We believe this strikes a good balance between readability and transparency, as the absolute misclassification counts provide useful insight into specific model errors. We hope this compromise is acceptable and improves the overall interpretability of the confusion matrices.

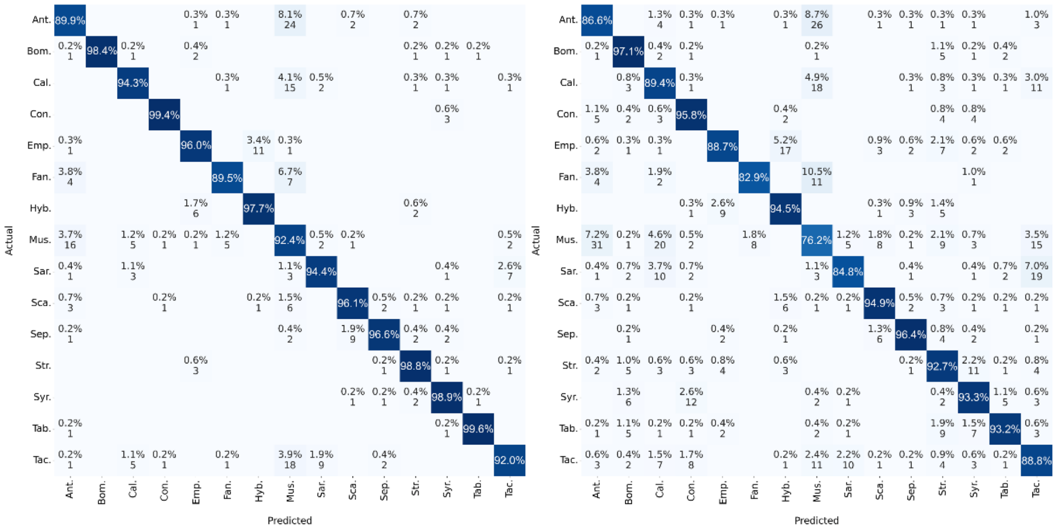


(a) Images cropped to bounding box

(b) Images not cropped to bounding box

- *The explanation of figure 5 is missing in this section.*

Figure 5 is intentionally not mentioned in the results section, as it is solely intended to support the discussion. In the discussion, we focus on confidence values for three Diptera families that exhibit notable morphological similarities. We believe this placement is appropriate, as it aligns with the interpretative nature of the discussion rather than the presentation of primary results.

## Discussion

- *Line 309: The sentence "The labor-intensive nature of this manual approach underscores the potential benefits of automation" seems to contradict the contribution of your work – I think a more interesting issue is the trade-off between the labour required to crop the images and the improvement in OA and mean confidence scores.*

Thank you for this valuable comment. We agree that the original sentence may have unintentionally emphasized automation at the expense of acknowledging the contribution of our manual cropping efforts. To better reflect the focus of our study, we revised the sentence to highlight the trade-off between the labor required for manual cropping and the observed improvements in overall accuracy and mean confidence. This adjustment aligns more closely with the contribution of our work and clarifies the practical relevance of the manual annotation process.

(L.374-382) Creating bounding boxes manually is an extremely time-consuming process. For the 29,374 images used in this study, bounding boxes had to be created by experts, following a consistent workflow to ensure that each specimen is cropped uniformly. This task requires not only significant time but also specialized expertise to accurately delineate the objects of interest. While the manual annotation process is undeniably labor-intensive, our results highlight a critical trade-off: the substantial effort required for cropping is justified by notable improvements in overall accuracy and mean confidence, suggesting that such manual intervention can significantly enhance model performance.

- *Figure 5: This figure is somewhat confusing – are the green boxes examples of two different species from the same family that have been correctly predicted? Further information in the legend would clarify this.*

Thank you for your helpful suggestion. Yes the green boxes are examples of two different species from the same family that have been correctly predicted. We have improved Figure 5 by adding clearer annotations that indicate both the true labels and the corresponding predictions, making the visualization easier to interpret. Additionally, we revised the figure caption to explicitly highlight these changes and to better guide the reader through the content of the figure.

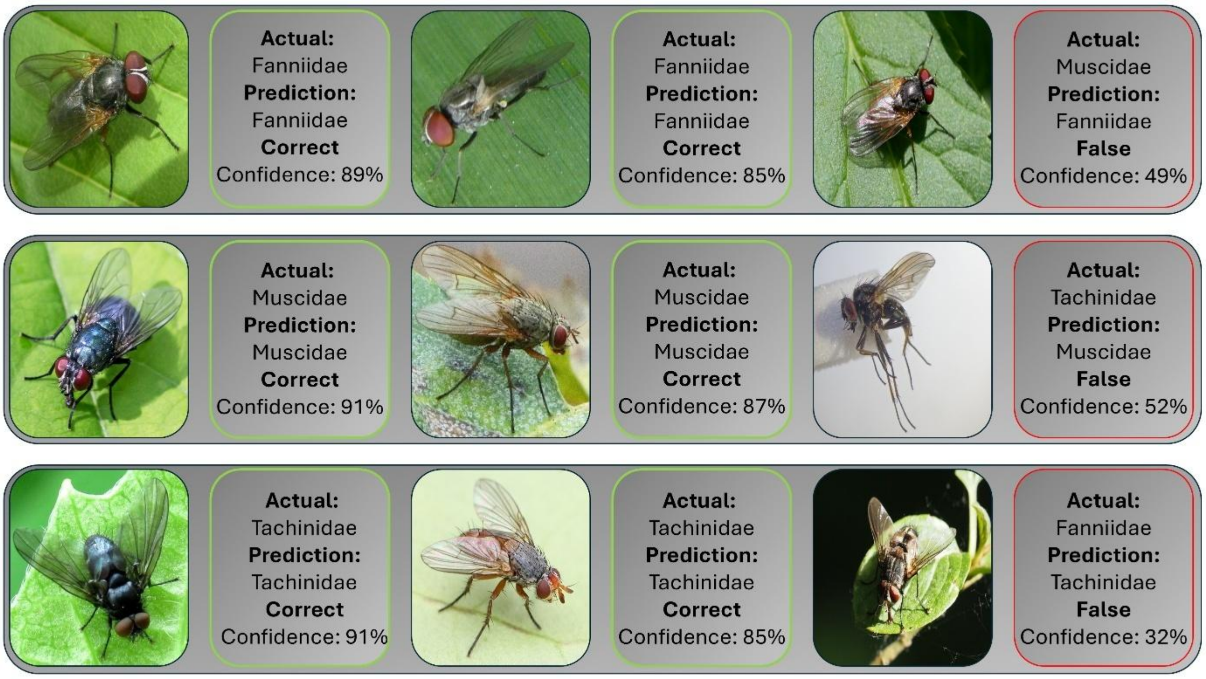


**Fig 5. Confidence values for three Diptera families with morphological similarities.** Predicted and actual labels for selected examples classified by EfficientNetB4 using images cropped to their bounding boxes. The model's predicted class, corresponding ground truth label, and prediction confidence are shown for each image.

- *Confusion between Diptera families: In the second and third paragraphs, the two families you are comparing Fanniidae and Muscidae have 521 and 2163 images respectively, what impact does this class imbalance between have on their OA and mean confidence?*

Thank you for raising this important point regarding class imbalance. Indeed, there is a notable difference in sample sizes between Fanniidae (521 images) and Muscidae (2163 images). While such imbalance can influence model performance, in this case the difference in classification accuracy is relatively small: 89.5% of Fanniidae and 92.4% of Muscidae images were correctly classified. It is possible that the smaller number of Fanniidae images (312 in the train set) limits the model's ability to generalize, and increasing this dataset could help improve performance.

Interestingly, the mean confidence values also reflect this trend. Predictions for Muscidae had a higher mean confidence of 0.89, whereas Fanniidae predictions averaged 0.78. This suggests that the model is not only more accurate for Muscidae, but also more certain in its classifications, likely benefiting from a larger and more representative training set. Nonetheless, the relatively high performance for both families indicates that the model is capable of distinguishing between them despite their morphological similarities and the class imbalance.

(L.455-466) From the confusion matrix in Figure 5.a, we observe that of the 105 Fannidae images in our test dataset, seven are misclassified as Muscidae. One such misclassification is shown in Figure 2a, where EfficientNet exhibits low confidence (49\%) in its classification. In contrast, correctly classified Fannidae images have higher confidence scores of 85\% and 89\%. Overall, the mean confidence for Fannidae predictions is 0.78.

For the Muscidae family, five of 433 test images are misclassified as Fannidae and two as Tachinidae. A misclassification of Tachinidae is depicted in Figure 5, with a low confidence of 52\%. Correct classifications for Muscidae have higher confidence scores of 87\% and 91\%. The mean confidence for Muscidae predictions is slightly higher at 0.89. This difference in mean confidence could be partially attributed to class imbalance, as the Fannidae family has significantly fewer training examples than Muscidae, potentially limiting the model's ability to generalize confidently.
